# Pain-related anxiety-like behavior requires CRF1 receptors in the amygdala

**DOI:** 10.1186/1744-8069-3-13

**Published:** 2007-06-05

**Authors:** Guangchen Ji, Yu Fu, Katherine A Ruppert, Volker Neugebauer

**Affiliations:** 1 Department of Neuroscience and Cell Biology, The University of Texas Medical Branch, Galveston, Texas 77555-1069, USA

## Abstract

Corticotropin-releasing factor receptor CRF1 has been implicated in the neurobiological mechanisms of anxiety and depression. The amygdala plays an important role in affective states and disorders such as anxiety and depression. The amygdala is also emerging as a neural substrate of pain affect. However, the involvement of the amygdala in the interaction of pain and anxiety remains to be determined. This study tested the hypothesis that CRF1 receptors in the amygdala are critically involved in pain-related anxiety. Anxiety-like behavior was determined in adult male rats using the elevated plus maze (EPM) test. The open-arm preference (ratio of open arm entries to the total number of entries) was measured. Nocifensive behavior was assessed by measuring hindlimb withdrawal thresholds for noxious mechanical stimulation of the knee. Measurements were made in normal rats and in rats with arthritis induced in one knee by intraarticular injections of kaolin/carrageenan. A selective CRF1 receptor antagonist (NBI27914) or vehicle was administered systemically (i.p.) or into the central nucleus of the amygdala (CeA, by microdialysis). The arthritis group showed a decreased preference for the open arms in the EPM and decreased hindlimb withdrawal thresholds. Systemic or intraamygdalar (into the CeA) administration of NBI27914, but not vehicle, inhibited anxiety-like behavior and nocifensive pain responses, nearly reversing the arthritis pain-related changes. This study shows for the first time that CRF1 receptors in the amygdala contribute critically to pain-related anxiety-like behavior and nocifensive responses in a model of arthritic pain. The results are a direct demonstration that the clinically well-documented relationship between pain and anxiety involves the amygdala.

## Background

Pain, including arthritis pain, has a negative affective component and is closely related to anxiety and depression [1-3]. The neural pathways and mechanisms involved in pain-related anxiety remain to be determined, but the amygdala is known to play a key role in emotional-affective behavior and anxiety disorders [4-6]. Importantly, the amygdala is emerging as an important element of the brain network involved in the emotional-affective component of pain [7-11]. The amygdala is also believed to be a key substrate of the reciprocal relationship between pain and affective states and disorders such as anxiety [3,10,12,13].

Our previous studies demonstrated central sensitization [14-19] and synaptic plasticity [14,20-23] in the central nucleus of the amygdala (CeA) in the kaolin/carrageenan-induced arthritis pain model. The CeA integrates affect-related information from the fear-anxiety circuitry in the lateral-basolateral amygdala with purely nociceptive inputs from the spino-parabrachio-amygdaloid pain pathway [7,9,10]. Pain-related synaptic plasticity in the CeA has also been confirmed in a model of chronic neuropathic pain [24]. It has become clear now that reversal of pain-related plasticity by pharmacologic deactivation of the CeA decreases nocifensive and affective pain responses in animal models of arthritic pain [14,25], visceral pain [26] and neuropathic pain [11] and in the prolonged phase of the formalin test [27].

The present study focused on the role of corticotropin-releasing factor receptor 1 (CRF1) in the CeA in pain-related anxiety. The CeA is a major site of extrahypothalamic expression of CRF and a key element of the extrahypothalamic circuits through which CRF contributes to anxiety-like behavior and affective disorders [28-32]. CRF1 receptors have emerged as drug targets for depression and anxiety disorders in preclinical studies [29-31,33-36]. A CRF1 receptor antagonist has been used successfully in humans to reduce depression and anxiety scores [37,38]. Finally, the presence of CRF-containing neurons in the parabrachial area [39] links the CRF system in the amygdala to the spino-parabrachio-amygdaloid pain pathway and implicates CRF in the transmission of nociceptive information to the amygdala.

## Findings

The behavioral and pharmacological studies reported here tested the hypothesis that CRF1 receptors in the amygdala (CeA) are critically involved in pain-related anxiety-like behavior. Adult male Sprague-Dawley rats (250–350 g) were used. All experimental procedures were approved by the Institutional Animal Care and Use Committee (IACUC) at the University of Texas Medical Branch (UTMB) and conform to the guidelines of the International Association for the Study of Pain (IASP) and of the National Institutes of Health (NIH).

**Figure 1:**
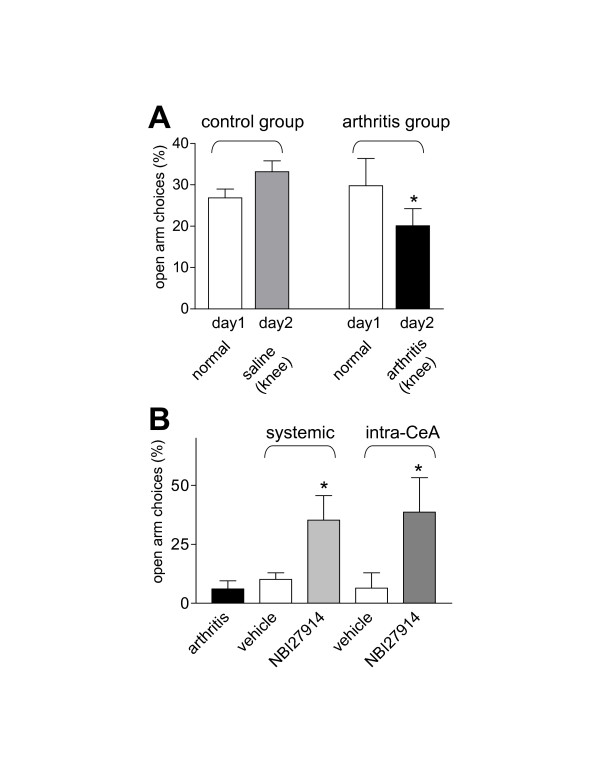
**Increased anxiety-like behavior in the arthritis pain model (A) is decreased by a CRF1 receptor antagonist (B)**. Anxiety-like behavior of adult male rats was determined by measuring the open-arm preference (ratio of open arm entries to the total number of entries expressed as %) in the elevated plus maze (EPM) test. **(A) **Open-arm choice did not change in control rats (n = 5) after intraarticular saline injections on day 2 compared to normal baseline on day 1. Rats with arthritis (n = 7; 5–6 h postinjection of kaolin/carrageenan into the knee on day 2) showed a significantly decreased open-arm preference compared to normal baseline on day 1 (P < 0.05, paired t-test), suggesting increased anxiety-like behavior. **(B) **A CRF1 receptor antagonist (NBI27914) administered systemically (5 mg/kg i.p.; n = 5) or into the CeA by microdialysis (100 μM, concentration in microdialysis fiber, 2 μl/min; n = 5) increased the open-arm preference significantly (P < 0.05, compared to vehicle groups; Newman-Keuls Multiple Comparison Test). Systemic (i.p.) application of saline (n = 5) or intra-amygdalar administration of ACSF (n = 5) as vehicle controls had no significant effect on open-arm choices compared to arthritic rats without any interventions (n = 6; P > 0.05; Newman-Keuls Multiple Comparison Test). Bar histograms show the mean ± SEM. * P < 0.05.

Anxiety-like behavior was determined using the elevated plus maze (EPM) test [40] (Figure 1). The open-arm preference (ratio of open arm entries to the total number of entries expressed as %) was measured for 45 min using a computerized recording and analysis system (Multi-Varimex v1.00; Columbus Instruments, OH, USA). Each rat was tested once on day 1 (normal baseline) and again on day 2 5–6 hours after intraarticular injection of sterile saline (control group) or kaolin and carrageenan (arthritis group) as previously described in detail [14,25,41,42]. In the control group (n = 5 rats), the percentage of open-arm choices (preference) was not significantly different between day 1 (normal baseline) and day 2 (intraarticular saline; P > 0.05, paired t-test; Fig. 1A). These data show that repeating the EPM test on day 2 does not per se alter baseline behavior. In the arthritis group (n = 7 rats), the open-arm preference decreased significantly (P < 0.05, paired t-test) 5–6 hours after arthritis induction (day 2) compared to normal baseline behavior (day 1), suggesting increased anxiety-like behavior (Fig. 1A).

Next we determined the effects of a selective CRF1 receptor antagonist (5-chloro-4-(N-(cyclopropyl)methyl-N-propylamino)-2-methyl-6-(2,4,6-trichlorophenyl) amino-pyridine, NBI 27914 [43]; purchased from Tocris Bioscience, Ellisville, MO). NBI27014 was administered either systemically (intraperitoneally, i.p.) or locally into the amygdala (CeA) by microdialysis in rats with arthritis (Fig. 1B). For drug application by microdialysis a guide cannula was implanted stereotaxically on the dorsal margin of the CeA as previously described in detail using the following coordinates [14,25]: 1.8–2.0 mm caudal to bregma, 4.0 mm lateral to midline, depth 7.0 mm. On the day of the experiment a microdialysis probe (CMA/Microdialysis 11; membrane diameter: 250 μm, membrane length: 2 mm) was inserted into the CeA through the guide cannula so that the probe protruded by 2 mm. The probe was connected to a Harvard infusion pump and perfused with ACSF (2 μl/min) for at least 1 h to establish equilibrium in the tissue.

Anxiety-like behavior was measured in 5 groups of rats to determine the role of CRF1 receptors: arthritic rats without any additional intervention; arthritic rats that received systemic administration of vehicle (saline); arthritic rats with systemic administration of NBI27014 (5 mg/kg, i.p.); arthritic rats with vehicle (ACSF) administration into the CeA; and arthritic rats with intra-CeA administration of NBI27014 (100 μM; concentration in microdialysis probe which is 100-fold that predicted to be needed based on data from our previous studies [15]). The open-arm preferences of arthritic rats without any interventions (n = 6) and of arthritis rats with systemic saline (n = 5) or intra-amygdala ACSF (n = 5) were not significantly different (P > 0.05, Newman-Keuls Multiple Comparison Test; GraphPad Prism software 3.0; Fig. 1B). Systemic application of NBI27914 30 min before the EPM test in increased the open-arm preference significantly (n = 5; P < 0.05, compared to saline group; Newman-Keuls Multiple Comparison Test). Administration of NBI27914 into the CeA for 30 min also increased the open-arm preference significantly (n = 5; P < 0.05, compared to ACSF control group; Newman-Keuls Multiple Comparison Test). The effects of systemic and intra-CeA administration of NBI27914 were not significantly different (P > 0.05; Newman-Keuls Multiple Comparison Test), suggesting that CRF1 receptors in the amygdala (CeA) account for the anxiolytic effect of CRF1 receptor antagonists.

**Figure 2:**
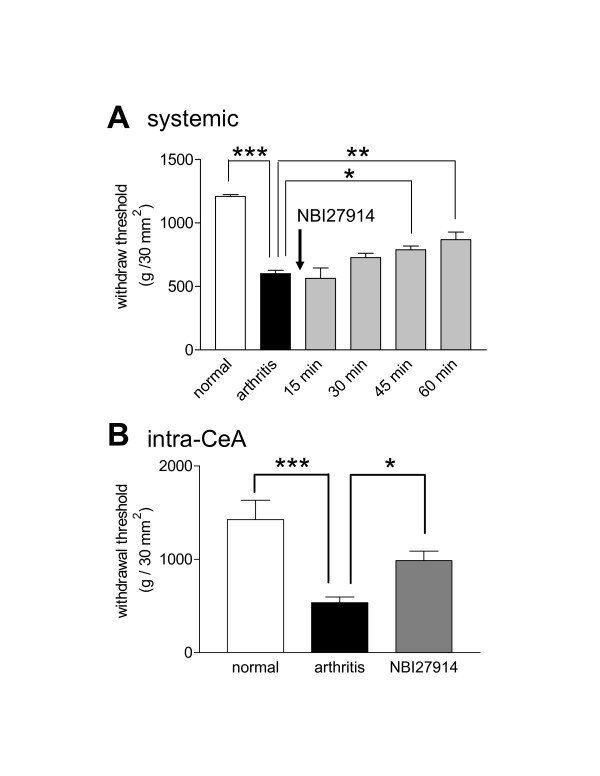
**Increased nocifensive behavior in the arthritis pain model is decreased by systemic (A) or intra-amygdala (B) administration of a CRF1 receptor antagonist**. Mechanical stimuli (compression) of continuously increasing intensity were applied to the knee joint of adult male rats to measure hindlimb withdrawal thresholds. **(A) **Thresholds decreased 5–6 h postinduction of arthritis in the knee by intraarticular injections of kaolin/carrageenan (n = 5; P < 0.001, repeated measures ANOVA followed by Newman-Keuls Multiple Comparison Test). Systemic administration of NBI27914 (5 mg/kg i.p.; n = 5) significantly increased the hindlimb withdrawal thresholds at 45 min (P < 0.05) and 60 min (P < 0.01) after drug injection (repeated measures ANOVA followed by Dunnett's posthoc test). **(B) **Intra-CeA administration of NBI27914 (100 μM; concentration in microdialysis fiber; n = 7) also increased the hindlimb withdrawal thresholds of arthritic rats significantly (P < 0.05; repeated measures ANOVA followed by Newman-Keuls Multiple Comparison Test). Bar histograms show the mean ± SEM. * P < 0.05.

We also determined the effects of a CRF1 antagonist on nocifensive responses (Fig. 2). Thresholds of hindlimb withdrawal reflexes evoked by mechanical stimulation of the knee joint were measured as previously described in detail [41,42]. Animals were paced in a custom-designed recording chamber that ensured stable and reproducible stimulations of the knee. Mechanical stimuli (compression) of continuously increasing intensity were applied to the knee joint using a calibrated forceps with a force transducer whose output was digitized and recorded on a computer. Measurements were made before (normal baseline) and 5–6 hours after arthritis induction. Arthritic rats received either systemic (n = 5; Fig. 2A) or intra-CeA (n = 7; Fig. 2B) administrations of NBI27914. Both groups of arthritic animals had significantly decreased hindlimb withdrawal thresholds indicating mechanical hypersensitivity (P < 0.001; repeated measures ANOVA followed by Newman-Keuls Multiple Comparison Test). Systemic administration of NBI27914 (5 mg/kg; n = 5) significantly increased the hindlimb withdrawal thresholds at 45 min (P < 0.05) and 60 min (P < 0.01) after i.p. injection (repeated measures ANOVA followed by Dunnett's posthoc test; Fig. 2A). Intra-CeA administration of NBI27914 (100 μM; concentration in microdialysis fiber; Fig. 2B) also increased the hindlimb withdrawal thresholds significantly (P < 0.05; repeated measures ANOVA followed by Newman-Keuls Multiple Comparison Test).

## Conclusion

In summary, this study showed that systemic or intra-amygdalar administration of a CRF1 receptor antagonist decreased anxiety-like behavior and nocifensive reflex responses in a model of arthritis pain, suggesting a key role of CRF1 receptors in the amygdala (CeA) in the modulation of pain-related anxiety. The novelty of this study is that it directly links the amygdala, through CRF1 receptors in the CeA, to pain-related anxiety, which is clinically well-documented but mechanistically not well understood.

Although the amygdala is known to play a key role in anxiety-like behavior through mechanisms that appear to involve CRF [28-32,44], its contribution to pain-related anxiety remains to be determined. Recent biochemical [45-47] and behavioral [48-51] studies point to the amygdala as an important site for the pain-modulatory effects of CRF. Increased expression of CRF1 receptor mRNA was detected in the amygdala in a model of somato-visceral pain induced by intra-peritoneal acetic acid [46]. CRF mRNA increased in the CeA in models of colitis pain [45] and chronic neuropathic pain [47]. Intracerebroventricular or intra-CeA administration of a broad-spectrum CRF receptor antagonist (alpha-hCRF9-41) had antinociceptive effects on hyperalgesic behavior associated with opiate withdrawal [50]. Systemic administration of a CRF1 receptor antagonist nearly reversed colon hypersensitivity (visceromotor response) induced by stereotaxic delivery of corticosterone to the CeA [49]. On the other hand, intra-CeA administration of a non-selective CRF receptor antagonist (alpha-hCRF9-41) produced hyperalgesic behavior (decreased mechanical and thermal withdrawal thresholds) and attenuated the antinociceptive effects of CRF administered into the CeA in normal animals [48]. The reason for these conflicting findings is unclear at this time. Our recent electrophysiological data show that administration of a CRF1 receptor antagonist (NBI27914) into the CeA clearly inhibits the sensitization of CeA neurons in the arthritis pain model. Taken together with the present study, these findings suggest that CRF1 receptors critically contribute to pain-related sensitization that results in increased pain responses and anxiety-like behavior.

## Authors' contributions

GJ and YF contributed equally to the paper by performing the experiments and data analysis. GJ provided the first draft of the manuscript. KAR also performed experiments and assisted with the data analysis. VN conceptualized the hypothesis, designed and supervised the experiments, directed the data analysis, and revised the manuscript. All authors read and approved the final manuscript.
